# Memorial of Joseph Murray

**Published:** 2013-01

**Authors:** Abdoljalil Kalantar-Hormozi, Ali Manafi

**Affiliations:** 1 Professor of Department of Plastic Surgery, Shahid Beheshti University of Medical Science, Tehran, Iran; 2 Editor-in-Chief of WJPS

It has not been too long since we realized that Joseph Murray (1919-2012) has passed away while we heard this sad news 2 weeks ago. We never forget his memories and trip for the first time on 14^th^ November 1974to Iran. He returned to our country one more time later to share his experiences with the country specialists. It is a great sorrow that he did not see our progress in plastic and reconstructive surgery now and how his dreams changed into reality. 

Joseph Murray, the world-known plastic surgeon and Nobel Prize winner, whose name embodies the honors of the first organ transplant surgeon, was invited to Iran in 1974 by Dr. Osanloo, the Head of the Department of Plastic Surgery of Tehran University. During his stay (From 14^th^ Nov to 12^th^ Dec 1974), he was involved in Iranian plastic surgeons training at the newly established, Motahari Burn and Plastic Surgery Center (Shahbanoo Hospital called at that time). 

Throughout the time he spent in Iran, he was busy in patient care and participating in the educational programs. He was also involved in designing the modern plastic surgery training in Iran organized by Dr. Osanloo while inviting the world well-known plastic surgeons such as Paul Tessier, Daniel O. Monasterio and Jaque Michon too. Having a world-class plastic surgery department in Iran was the dream of Dr Murray’s and Dr Osanaloo in Iran which changed into reality by his efforts. 

Upon his arrival in the United States of America, he surveyed the strength and weaknesses points of the Iranian plastic surgery program and wrote his report to Dr. Osnaloo in this relation. This report had a great influence on the scientific foundation of plastic surgery program in Iran which now exists. 

Part of his report is as follows:

**Harvard Medical School Surgery Unit, Plastic Surgery Department Report**

**Children Hospital, Joseph Murray, MD, Professor of Surgery**

**Peter Bent Brigham Hospital, 721 Huntington Ave. Boston, MA, 2115**

**To: Sirous Osanloo, MD,**

**From: Joseph Murray, MD,**

**6/01/1975**

***Memorandom***

**Fig. 1:**
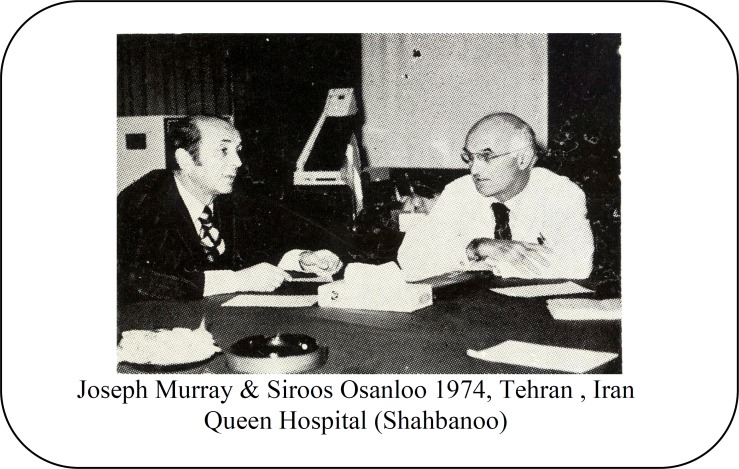
Joseph Murray and Sirous Osanloo in their meeting in 1974. Extracted from Shahbanoo Hospital Newsletter

I doubtfully arrived at Shahbanoo Hospital while returned from there with strong confidence and beliefs while I believed in Dr. Osanloo’s opinions that he would have a high standard treatment unit for Iranian patients. Beside to Dr. Osanloo’s knowledge and beliefs on medical issues and his sociopolitical rank in Iran, his decision to have a standard patient care was worthy resulted into establishment of the Shahbanoo Hospital. This hospital provided the best services for the Iranian patients and also for all other nations too. 

**Fig. 2:**
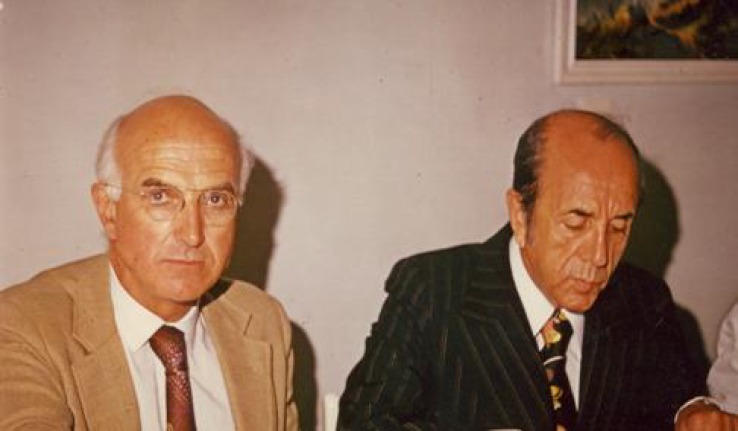
Dining ceremony in Shahbanoo Hospital. Left is Dr. J. Murray and right Dr. S. Osanloo (With permission from AIM Journal).

Although, his action can not be extendable throughout the world, but it was a good choice for Iranian patients. So I would like to have a close and sincere cooperation with him enthusiastically. I believe that his opinion on inviting the world famous specialists for training, consulting and working in this hospital can be the most beneficial activity and a wise decision. 

In training programs, one cannot rely just on the guest professors. Hence, it is worthwhile to create a solid core of intelligent and well-educated Iranian physicians who have a reasonable income for a better medical service to their people. I can truly say that I entered Iran just as a foreigner but left the country with sweet memories of good friends and colleagues that I would never forget them.

I sincerely wish that I could return again to Iran soon. But as I said before, I cannot make this decision sooner than three or four months. This is due to the duties and responsibilities that I deal with in Harvard University, Boston, as I should be there as a full time faculty member soon.

**Sincerely Yours**

**Joseph Murray, MD,**

**06/01/1975**

**Boston** **,** **MA** **,** **USA** **.**
